# Centenary of the Brazilian Nursing Association: struggles, challenges, and achievements

**DOI:** 10.1590/0034-7167.20267905

**Published:** 2026-08-17

**Authors:** Jacinta de Fatima Sena da Silva, Maria Helena Palucci Marziale, Dulce Aparecida Barbosa, Antonio José de Almeida

**Affiliations:** I President of the Brazilian Nursing Association. Brasília, Federal District, Brazil; II Full Professor, Escola de Enfermagem de Ribeirão Preto, Universidade de São Paulo. Director of Publications of the Brazilian Nursing Association. Ribeirão Preto, São Paulo, Brazil; III Universidade Federal de São Paulo, Escola Paulista de Enfermagem. Editor-in-Chief of the Brazilian Journal of Nursing. São Paulo, São Paulo, Brazil; IV Universidade Federal do Rio de Janeiro, Escola de Enfermagem Anna Nery. Scientific Editor of the Brazilian Journal of Nursing. Rio de Janeiro, Rio de Janeiro, Brazil

The Brazilian Nursing Association (ABEn - *Associação Brasileira de Enfermagem*), founded on August 12, 1926, marked a turning point in the history of Brazilian nursing. ABEn emerged as a collective project aimed at building nursing’s scientific sovereignty, aligned with the struggle for the social determination of health and the defense of the profession’s ontological identity. Thus, as we celebrate the 100 years of ABEn’s activities, we engage not only in an important institutional celebration but also in critical reflections on the association’s role in professional education, Brazilian nursing science, and society. Its active participation in political advocacy efforts relevant to nursing and its contribution to the country’s health and education policies deserve special recognition.

Throughout its centenary, ABEn has served as a transformative agent in the field of professional education, participating in the development of educational policies aimed at the qualification and recognition of nursing professionals. The institution has acted as a mediator between theory and practice, promoting the scientific and social praxis of nursing within the context of the Unified Health System (SUS - *Sistema Único de Saúde*). In this regard, ABEn has demonstrated its commitment to the social determination of health, advocating for the centrality of care and equity in access to healthcare services. Its leadership in the movement to review and update the National Curriculum Guidelines^(1)^ for Undergraduate Nursing Education, recently approved, deserves particular emphasis. This action was fundamental to ensuring an in-person, ethical, and socially committed educational model, reaffirming the defense of education and the SUS as public goods.

Among the many accomplishments of this centennial professional association, its commitment to preserving nursing memory stands out, a concern shared by every ABEn administration since its foundation. The extensive documentary collection produced, preserved, and organized over the past 100 years reflects the dedication of its presidents, boards of directors, and countless collaborators and researchers, constituting a true institutional heritage.

In this regard, special mention should be made of the documentary entitled “Brazilian Nursing Association: 1926-1976” (*Associação Brasileira de Enfermagem: 1926-1976*), published in 1976 by Anayde Corrêa de Carvalho, as well as the consolidation of the Brazilian Nursing Memory Center (CEMEnf - *Centro de Memória da Enfermagem Brasileira*), inaugurated on August 4, 2010, at the headquarters of ABEn Nacional in Brasília, Federal District. CEMEnf serves as an important research laboratory for historians in the fields of health and nursing^(2)^.

In this sense, CEMEnf should be understood as a heritage of both material and symbolic nature. From this perspective, historical archives are fundamental epistemic instances for the analysis and understanding of past events that constitute the identity of nursing. The Department of Nursing History (DHE - *Departamento Científico de História da Enfermagem*) of ABEn *Nacional*, established in 2010, represents yet another investment by the Association in the preservation and mapping of the material and intangible heritage of Brazilian nursing^(3)^. Another major initiative, launched in 2025 during the 75^th^ Brazilian Nursing Congress (CBEn - *Congresso Brasileiro de Enfermagem*), held in November of that year in Porto Alegre, Rio Grande do Sul, entitled “ABEn House: Memorial of Brazilian Nursing” (*Casa ABEn: memorial da enfermagem brasileira*), consists of a virtual metamemorial of the association and represents another effort to disseminate the history of nursing through ABEn. This project allows researchers, students, and society to access the association’s collection, aiming to promote the appreciation and preservation of nursing history and professional identity.

Another area of investment by the Association concerns the production and dissemination of knowledge. Special recognition must therefore be given to the creation of the Brazilian Journal of Nursing (REBEn - *Revista Brasileira de Enfermagem*) in 1932, which celebrates 94 years of existence in 2026. It is worth emphasizing that the creation of REBEn and the Brazilian National Nursing Congress (1947) not only enabled better communication among nurses but also represented the first intellectual environments for these professionals. The first Brazilian National Nursing Congress, as it was originally named, was held at the *Universidade de São Paulo* School of Nursing from March 18 to 22, 1947. In its inaugural edition, the event demonstrated the concern of its organizers with fostering a collective effort toward the advancement of Brazilian nursing^(4,5)^.

On the eve of the establishment of the first master’s degree program in nursing in the country, the proposal to create a research center specifically dedicated to Brazilian nursing gained momentum. This initiative gave rise to the Center for Nursing Studies and Research (CEPEn - *Centro de Estudos e Pesquisas em Enfermagem*), officially established during the Assembly of Delegates held in Rio de Janeiro in 1976, on the occasion of the approval of the reform of ABEn’s statutes^(6)^. CEPEn organized its first research seminar in 1979, called the National Nursing Research Seminar (SENPE - *Seminário Nacional de Pesquisa em Enfermagem*)^(3,6)^.

Other thematic events were created during the 1990s and 2000s. In 1994, ABEn promoted the first Brazilian National Seminar on Guidelines for Nursing Education; in 1991, the Brazilian National Symposium on Nursing Diagnosis; in 2003, the International Seminar on Nursing Work; in 2007, the Brazilian National Seminar on Guidelines for Nursing in Primary Health Care; and the Latin American Colloquium on Nursing History, which has been affiliated with ABEn since 2012. In 2025, its seventh edition was held, and, through the initiative of leaders involved in scientific publishing in nursing, the Forum of Nursing Journal Editors was permanently established alongside CBEns and SENPEs, having already held 26 editions^(3)^.

The creation of the DHE demonstrates and reaffirms the association’s commitment to preserving institutional memory and the history of Brazilian nursing. Researchers involved with this department conceived and created, under the auspices of ABEn, the History of Nursing: Electronic Journal (*História da Enfermagem: Revista Eletrônica*), a journal dedicated to the history of nursing and health.

Numerous achievements have been promoted, sponsored, and led by ABEn across different spheres, including science, education, social assistance, culture, and politics, extending far beyond its physical headquarters. This thematic issue of REBEn is part of the commemorative activities celebrating ABEn, which, in its centenary year, demonstrates the vitality necessary to reach many more centenaries. Congratulations and long life to our beloved ABEn.
