# Supplementary material for: Centenary of the Brazilian Nursing Association: struggles, challenges, and achievements
Source: Rev Bras Enferm. 2026 Aug 17;79:e7905. doi: 10.1590/0034-7167.20267905 (PMC13481039; doi:10.1590/0034-7167.20267905)
Supplement: Translation

# Translation: Centenário da Associação Brasileira de Enfermagem: lutas, desafios e conquistas

**DOI:** 10.1590/0034-7167.20267905pt

**Authors:** Jacinta de Fatima Sena da Silva, Maria Helena Palucci Marziale, Dulce Aparecida Barbosa, Antonio José de Almeida

**Affiliations:** I Presidenta da Associação Brasileira de Enfermagem. Brasília, Distrito Federal, Brasil; II Professora Titular da Escola de Enfermagem de Ribeirão Preto da Universidade de São Paulo. Diretora de Publicações da Associação Brasileira de Enfermagem. Ribeirão Preto, São Paulo, Brasil; III Universidade Federal de São Paulo, Escola Paulista de Enfermagem. Editora-Chefe da Revista Brasileira de Enfermagem. São Paulo, São Paulo, Brasil; IV Universidade Federal do Rio de Janeiro, Escola de Enfermagem Anna Nery. Editor Científico da Revista Brasileira de Enfermagem. Rio de Janeiro, Rio de Janeiro, Brasil

A Associação Brasileira de Enfermagem (ABEn), criada em 12 de agosto de 1926, marcou um divisor de águas na história da enfermagem brasileira. A ABEn emergiu como um projeto coletivo de construção da soberania científica da enfermagem, alinhada à luta pela determinação social da saúde e à defesa da identidade ontológica da categoria. Assim, ao comemorarmos os 100 anos de atividades da ABEn, fazemos não só uma importante celebração institucional, mas também reflexões críticas sobre o papel da associação para a formação profissional, para a ciência da enfermagem brasileira e para a sociedade. Destaca-se a sua ativa participação em reivindicações políticas de interesse da enfermagem e a sua contribuição para as políticas de saúde e educação do país.

A ABEn, ao longo de seu centenário, tem sido um agente transformador no campo da formação profissional, participando da construção de políticas educativas que visam à qualificação e à valorização dos profissionais de enfermagem. A instituição tem atuado como mediadora entre a teoria e a prática, promovendo a práxis científica e social da enfermagem no contexto do Sistema Único de Saúde (SUS). Nesse sentido, a ABEn tem se mostrado uma entidade comprometida com a determinação social da saúde, defendendo a centralidade do cuidado e a equidade no acesso aos serviços de saúde. Cabe destacar a sua liderança diante do movimento de revisão e atualização das Diretrizes Curriculares Nacionais^(1)^ da Graduação em Enfermagem, recentemente homologadas. Essa ação foi fundamental para assegurar uma formação presencial, ética e socialmente comprometida, reafirmando a defesa da educação e do SUS como bens públicos.

Entre outros grandes feitos dessa centenária entidade associativa, destacamos seu interesse pela preservação da memória da enfermagem, objeto de preocupação de todas as gestões da ABEn desde a sua criação. O vasto acervo documental produzido, preservado e organizado ao longo de 100 anos reflete o compromisso das presidentes, diretorias e inúmeros colaboradores e pesquisadores, constituindo um verdadeiro patrimônio institucional.

Nesse sentido, destacamos o documentário intitulado “Associação Brasileira de Enfermagem: 1926-1976”, publicado em 1976 por Anayde Corrêa de Carvalho, e a consolidação do Centro de Memória da Enfermagem Brasileira (CEMEnf), inaugurado em 4 de agosto de 2010 na sede da ABEn Nacional, em Brasília, Distrito Federal. O CEMEnf funciona como um importante laboratório de pesquisa para historiadores das áreas da saúde e da enfermagem^(2)^.

Nesse sentido, o CEMEnf deve ser compreendido como um patrimônio de natureza material e simbólica. Nessa perspectiva, os arquivos históricos são instâncias epistêmicas fundamentais para a análise e a compreensão dos eventos pretéritos que constituem a identidade da enfermagem. O Departamento Científico de História da Enfermagem (DHE) da ABEn Nacional, criado em 2010, é mais um investimento da associação na guarda e no mapeamento do patrimônio material e imaterial da enfermagem brasileira^(3)^. Outro projeto grandioso, lançado em 2025, no âmbito do 75º Congresso Brasileiro de Enfermagem (CBEn), que ocorreu em novembro do referido ano em Porto Alegre, estado do Rio Grande do Sul, intitulado “Casa ABEn: memorial da enfermagem brasileira”, trata de um metamemorial virtual da associação, sendo mais uma iniciativa para divulgar a história da enfermagem através da ABEn. Esse projeto permite que pesquisadores, estudantes e a sociedade acessem o acervo da associação, visando à valorização e à preservação da história e da identidade profissional.

Outra frente de investimento da associação refere-se à produção e à difusão do conhecimento. Portanto, um destaque especial vai para a criação da Revista Brasileira de Enfermagem (REBEn), em 1932, concluindo 94 anos de existência em 2026. Vale ressaltar que a criação da REBEn e o Congresso Nacional de Enfermagem (1947), além de permitirem uma melhor comunicação entre as enfermeiras, também representaram os primeiros ambientes intelectuais dessas profissionais. O primeiro Congresso Nacional de Enfermagem, como foi inicialmente denominado, ocorreu nas dependências da Escola de Enfermagem da Universidade de São Paulo no período de 18 a 22 de março de 1947. O evento, em sua primeira versão, demonstrava a preocupação de suas organizadoras em uma construção coletiva em prol dos avanços da enfermagem brasileira^(4,5)^.

Às vésperas da criação do primeiro mestrado em enfermagem no país, ganha robustez a proposta de criação de um centro de pesquisas voltado especificamente à enfermagem brasileira. Tal iniciativa deu origem ao Centro de Estudos e Pesquisas em Enfermagem (CEPEn), cuja implantação oficial ocorreu em Assembleia de Delegados, realizada no Rio de Janeiro, em 1976, por ocasião da aprovação da reforma dos estatutos da ABEn^(6)^. O CEPEn realizou o primeiro seminário de pesquisa, em 1979, denominado Seminário Nacional de Pesquisa em Enfermagem (SENPE)^(3,6)^.

Outros eventos temáticos foram criados nas décadas de 1990 e 2000. A ABEn promoveu, em 1994, o primeiro Seminário Nacional de Diretrizes para a Educação em Enfermagem; em 1991, o Simpósio Nacional de Diagnóstico de Enfermagem; em 2003, o Seminário Internacional sobre o Trabalho na Enfermagem; em 2007, o Seminário Nacional de Diretrizes para Enfermagem na Atenção Básica em Saúde; o Colóquio Latino-Americano de História da Enfermagem, em sua terceira versão, vinculado à ABEn desde 2012. Em 2025, foi realizada a 7ª versão, e por iniciativa das lideranças envolvidas com a editoração científica na enfermagem, criou-se permanentemente o Fórum de Editores de Periódicos de Enfermagem, junto aos CBEns e aos SENPEs, com 26 versões realizadas^(3)^.

A criação do DHE evidencia e ratifica o comprometimento da associação com a preservação da memória institucional e a história da enfermagem brasileira. Os pesquisadores envolvidos com esse departamento idealizaram e criaram, sob os auspícios da ABEn, a História da Enfermagem: Revista Eletrônica, um periódico dedicado à história da enfermagem e da saúde.

São inúmeros os feitos promovidos, patrocinados e liderados pela ABEn em diferentes âmbitos: ciência, educação, assistência social, cultura e política, para além desse espaço físico. Esse número temático da REBEn integra as atividades comemorativas da ABEn, que, em seu centenário, demonstra o fôlego necessário para outros centenários. Parabéns e vida longa à nossa querida ABEn.

# Translation: Centenario de la Asociación Brasileña de Enfermería: luchas, desafíos y conquistas

**DOI:** 10.1590/0034-7167.20267905es

**Authors:** Jacinta de Fatima Sena da Silva, Maria Helena Palucci Marziale, Dulce Aparecida Barbosa, Antonio José de Almeida

**Affiliations:** I Presidenta de la Asociación Brasileña de Enfermería. Brasília, Distrito Federal, Brasil; II Profesora Titular de la Escola de Enfermagem de Ribeirão Preto de la Universidade de São Paulo. Directora de Publicaciones de la Asociación Brasileña de Enfermería. Ribeirão Preto, São Paulo, Brasil; III Universidade Federal de São Paulo, Escola Paulista de Enfermagem. Editora Jefe de la Revista Brasileña de Enfermería. São Paulo, São Paulo, Brasil; IV Universidade Federal do Rio de Janeiro, Escola de Enfermagem Anna Nery. Editor Científico de la Revista Brasileña de Enfermería. Rio de Janeiro, Rio de Janeiro, Brasil

La Asociación Brasileña de Enfermería (ABEn - *Associação Brasileira de Enfermagem*), creada el 12 de agosto de 1926, marcó un punto de inflexión en la historia de la enfermería brasileña. La ABEn surgió como un proyecto colectivo de construcción de la soberanía científica de la enfermería, alineado con la lucha por la determinación social de la salud y la defensa de la identidad ontológica de la categoría profesional. Así, al conmemorar los 100 años de actividades de la ABEn, realizamos no solo una importante celebración institucional, sino también reflexiones críticas sobre el papel de la asociación en la formación profesional, en la ciencia de la enfermería brasileña y en la sociedad. Se destaca su activa participación en reivindicaciones políticas de interés para la enfermería y su contribución a las políticas de salud y educación del país.

A lo largo de su centenario, la ABEn ha sido un agente transformador en el ámbito de la formación profesional, participando en la construcción de políticas educativas orientadas a la cualificación y valorización de los profesionales de enfermería. La institución ha actuado como mediadora entre la teoría y la práctica, promoviendo la praxis científica y social de la enfermería en el contexto del Sistema Único de Salud (SUS - *Sistema Único de Saúde*). En este sentido, la ABEn se ha mostrado comprometida con la determinación social de la salud, defendiendo la centralidad del cuidado y la equidad en el acceso a los servicios de salud. Cabe destacar su liderazgo en el movimiento de revisión y actualización de las Directrices Curriculares Nacionales^(1)^ de la Carrera de Enfermería, recientemente homologadas. Esta acción fue fundamental para garantizar una formación presencial, ética y socialmente comprometida, reafirmando la defensa de la educación y del SUS como bienes públicos.

Entre los numerosos logros de esta entidad asociativa centenaria, destaca su interés por la preservación de la memoria de la enfermería, una preocupación presente en todas las gestiones de la ABEn desde su creación. El vasto acervo documental producido, preservado y organizado a lo largo de 100 años refleja el compromiso de sus presidentas, directorios y de innumerables colaboradores e investigadores, constituyendo un verdadero patrimonio institucional.

En este sentido, se destaca el documental titulado “Asociación Brasileña de Enfermería: 1926-1976” (*Associação Brasileira de Enfermagem: 1926-1976*), publicado en 1976 por Anayde Corrêa de Carvalho, así como la consolidación del Centro de Memoria de la Enfermería Brasileña (CEMEnf - *Centro de Memória da Enfermagem Brasileira*), inaugurado el 4 de agosto de 2010 en la sede de la ABEn Nacional, en Brasília, Distrito Federal. El CEMEnf funciona como un importante laboratorio de investigación para historiadores de las áreas de la salud y de la enfermería^(2)^.

El CEMEnf debe entenderse como un patrimonio de naturaleza material y simbólica. Desde esta perspectiva, los archivos históricos constituyen instancias epistémicas fundamentales para el análisis y la comprensión de los acontecimientos pasados que conforman la identidad de la enfermería. El Departamento Científico de Historia de la Enfermería (DHE - *Departamento Científico de História da Enfermagem*) de la ABEn Nacional, creado en 2010, representa una inversión adicional de la Asociación en la preservación y el mapeo del patrimonio material e inmaterial de la enfermería brasileña^(3)^. Otro proyecto de gran relevancia, lanzado en 2025 durante el 75º Congreso Brasileño de Enfermería (CBEn - *Congresso Brasileiro de Enfermagem*), realizado en noviembre de ese año en Porto Alegre, estado de Rio Grande do Sul, titulado “Casa ABEn: memorial de la enfermería brasileña” (*Casa ABEn: memorial da enfermagem brasileira*), consiste en un metamemorial virtual de la asociación y constituye una iniciativa más para difundir la historia de la enfermería a través de la ABEn. Este proyecto permite que investigadores, estudiantes y la sociedad accedan al acervo de la asociación, con el objetivo de valorar y preservar la historia y la identidad profesional.

Otra área de inversión de la asociación se refiere a la producción y difusión del conocimiento. Por ello, merece especial destaque la creación de la Revista Brasileña de Enfermería (REBEn - *Revista Brasileira de Enfermagem*), en 1932, que cumple 94 años de existencia en 2026. Cabe señalar que la creación de la REBEn y del Congreso Nacional de Enfermería (1947), además de permitir una mejor comunicación entre las enfermeras, representó también los primeros espacios intelectuales para estas profesionales. El primer Congreso Nacional de Enfermería, como fue denominado inicialmente, tuvo lugar en las dependencias de la Escuela de Enfermería de la *Universidade de São Paulo* entre los días 18 y 22 de marzo de 1947. En su primera edición, el evento reflejaba la preocupación de sus organizadoras por una construcción colectiva orientada al avance de la enfermería brasileña^(4,5)^.

En vísperas de la creación de la primera maestría en enfermería del país, cobró fuerza la propuesta de establecer un centro de investigación dedicado específicamente a la enfermería brasileña. Esta iniciativa dio origen al Centro de Estudios e Investigaciones en Enfermería (CEPEn - *Centro de Estudos e Pesquisas em Enfermagem*), cuya implantación oficial tuvo lugar en una Asamblea de Delegados realizada en Río de Janeiro en 1976, con motivo de la aprobación de la reforma de los estatutos de la ABEn^(6)^. El CEPEn realizó su primer seminario de investigación en 1979, denominado Seminario Nacional de Investigación en Enfermería (SENPE - *Seminário Nacional de Pesquisa em Enfermagem*)^(3,6)^.

Otros eventos temáticos fueron creados durante las décadas de 1990 y 2000. La ABEn promovió, en 1994, el primer Seminario Nacional de Directrices para la Educación en Enfermería; en 1991, el Simposio Nacional de Diagnóstico de Enfermería; en 2003, el Seminario Internacional sobre el Trabajo en Enfermería; en 2007, el Seminario Nacional de Directrices para la Enfermería en la Atención Primaria de Salud; y el Coloquio Latinoamericano de Historia de la Enfermería, vinculado a la ABEn desde 2012. En 2025 se celebró su séptima edición y, por iniciativa de los líderes involucrados en la edición científica de enfermería, se creó de forma permanente el Foro de Editores de Revistas de Enfermería, junto a los CBEns y SENPEs, con 26 ediciones realizadas^(3)^.

La creación del DHE evidencia y ratifica el compromiso de la asociación con la preservación de la memoria institucional y de la historia de la enfermería brasileña. Los investigadores vinculados a este departamento concibieron y crearon, bajo los auspicios de la ABEn, la Historia de la Enfermería: Revista Electrónica (*História da Enfermagem: Revista Eletrônica*), una revista dedicada a la historia de la enfermería y de la salud.

Son innumerables los logros promovidos, patrocinados y liderados por la ABEn en diferentes ámbitos: ciencia, educación, asistencia social, cultura y política, trascendiendo este espacio físico. Este número temático de la REBEn forma parte de las actividades conmemorativas de la ABEn, que, en su centenario, demuestra el vigor necesario para alcanzar muchos otros centenarios. Felicitaciones y larga vida a nuestra querida ABEn.
